# Suffering for Water, Suffering from Water: Access to Drinking-water and Associated Health Risks in Cameroon

**DOI:** 10.3329/jhpn.v28i5.6150

**Published:** 2010-10

**Authors:** H. Blaise Nguendo Yongsi

**Affiliations:** Department of Human Sciences and Nursing University of Chicoutimi, 555 Boulevard de l'Universite, Québec G7h 2B1, Canada

**Keywords:** Cross-sectional studies, Diarrhoea, Disparity, Drinking-water, Risk factors, Water quality, Water pollution, Water supply, Cameroon

## Abstract

Although many African countries, along the equator, receive a great amount of rainfall and possess a dense hydrographic network, access to drinking-water remains a great challenge. In many households, water is used for various purposes, including domestic and crafts activities. According to the World Health Organization, an estimated four billion cases of diarrheoa occurs worldwide, of which 88% are ascribed to unsafe drinking-water. This study aimed at evaluating health risks in the usage of contaminated drinking-water and its relationship with the prevalence of diarrhoeal diseases in Yaoundé, Cameroon. In this cross-sectional epidemiological design, 3,034 households with children aged less than five years were investigated. Households were selected from among 20 representative neighbourhoods out of 105 that made up the city. The study revealed a diarrheoa prevalence of 14.4% (437 diarrheoa cases out of 3,034 children tested). Among various risk factors examined, water-supply modes and quality of drinking-water were statistically associated with diarrheoa cases. Moreover, levels of diarrheoa attacks varied considerably from one neighbourhood to the other. The spatial analysis helped determine neighbourhoods of higher and lower prevalence of diarrheoa in the city.

## INTRODUCTION

The availability of safe drinking-water is an increasing major concern for the interventional community, especially in light of changing climate-depleting biodiversity. Access to safe drinking-water for domestic use has become a major challenge for contemporary societies with its increased demand. Demand for clean and safe water has become more acute in the context of growing global population, particularly in less-developed countries (1). While developed countries invest heavily on the supply of fresh water to their entire population, developing countries are struggling to cater to the water needs of their citizens, especially in the context of rapid population growth and urbanization. Hence, urbanization has been phenomenal and puzzling with a rapid shift from 15% in 1950 to about 41% in 2007. It is estimated that, by 2030, the continent may attain 54% of urban proportion (2). This phenomenal growth has been qualified as sudden and wild to express the uncontrolled nature of urban growth and the implications it may have on the well-being of city-dwellers. Sub-Saharan Africa is ranked among the world's regions which are mostly at a disadvantage. It is confronted with acute ‘water problems’ (a threat in water shortage in sufficient and satisfactory quantity for human needs), which has negative impacts on a large number of people. It is estimated that close to 300 million people do not have access to drinking-water (3). Results of research showed that water used in most households in developing countries are unsafe for consumption (4–6). It is also evident that, each year, contaminated drinking-water contributes to the death of millions of the poorest people of the world from preventable diseases (7). More importantly, vulnerable groups, such as children, women, and the elderly, are the main victims. Empirical evidence also shows the nexus sanitation, polluted drinking-water, and health. In particular, two types of relationship are often stressed. First, contamination by human or animal faeces is the most regular and pervasive health risk associated with drinking-water; when such defect is recent and when those responsible for it include carriers of communicable enteric diseases, microorganisms that cause these diseases may be present in the water. Second, contaminated drinking-water may result in waterborne diseases, such as cholera, dysentery, and other diseases that may cause diarrhoeas (8).

Globally, it is estimated that 88% of diarrheoal disease cases are attributable to unsafe water. In fact, the World Health Organization (WHO) estimates that about 1.1 billion people globally drink unsafe water (9,10). According to the WHO/United Nations Children's Fund, diarrheoal diseases account for 4.3% of the total global burden of disease (62.5 million disability-adjusted life-years) (11). Despite the number of studies carried out, relatively little is known about the key contribution of unsafe drinking (reference is made here to pathogens found in water) in the occurrence of diarrhoeal diseases. Among the regions of the world, sub-Saharan Africa has been poorly covered despite being the fastest-growing urban population and the majority of city-dwellers having the least access to urban infrastructure and services. Within the context of cities in Cameroon which are witnessing constant population growth (12), access to water through taps is a luxury which only a few inhabitants can afford. With the population growth and the urban sprawling, connecting running water throughout the city requires expanding the water supply network, which the city councils and the government cannot afford. Therefore, many urban dwellers resort to various water sources of poor quality. Unsafe water is often contaminated with faecal material, domestic and industrial wastes. Such polluted water results in an increased risk of transmission of disease to individuals (13). Diarrhoeal diseases are often caused by contaminated water, poor sanitation, and poor hygiene. In Cameroon, diarrhoeal diseases are the most prevalent waterborne diseases among children aged less than five years. In Yaoundé, for example, the prevalence of diarrhoea is increasing. Results of studies conducted in the city among children aged less than five years showed that the rate of prevalence increased from 10.8% in 1998 to 13.1% in 2004 (14).

Epidemiological investigations can provide strong evidence linking exposure to the occurrence of diseases in a population and also estimate the magnitude of risk related to a particular exposure. This study, therefore, sought to assess the problem of access to drinking-water in Yaoundé. The objectives were three-fold: (a) to examine patterns of water supply in the Cameroonian capital to illustrate the complexity of the situation; (b) to assess the microbial quality of water used for consumption so as to evaluate its implication on the occurrence of diarrhoeal diseases; and (c) to gain an understanding of the geographic variations or patterns based on the prevalence of diagnosed morbidity.

## MATERIALS AND METHODS

### Study area and sampling sites

**Fig. 1.**
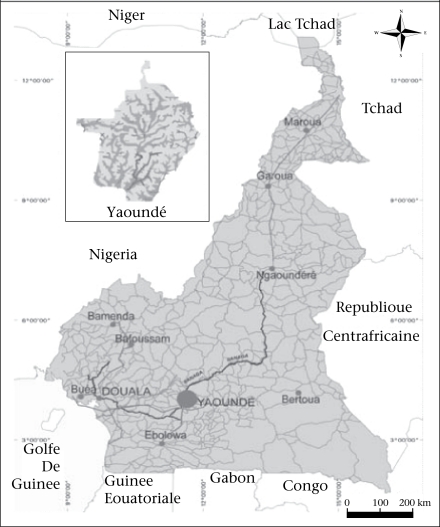
Map showing location of Yaoundé in Cameroon

**Fig. 2.**
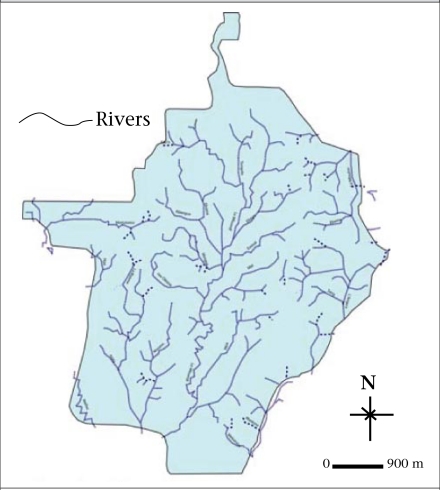
The hydrographic (drainage) network

Source: Yaounde topographic sheet no. Na-32-XXIV, 1962 Designing: H.B. Nguendo Youngsi, 2009 Software: Adobe Illustrator 11.0, Corel Draw 9.1

The study was conducted in Yaoundé, the capital city of Cameroon, situated in Central Africa, between latitudes 3 °47’ and 3 °56’ North and 11 °10’ and 11 °45’ East (Fig. 1). Yaoundé displays the classical equatorial climate, with regular and abundant rainfall of more than 1,600 mm per annum and a fairly high average annual temperature of 23 °C. Divided into four watershed basins, the Yaoundé watershed network is dense. The city is drained by the Mfoundi river and its many tributaries (Fig. 2). Like many sub-Saharan African cities, Yaoundé is currently experiencing very rapid urbanization. The first population census in 1926 estimated that Yaoundé had 100,000 inhabitants. With an estimated annual growth rate of 4.5% since 1980, its population has grown from 812,000 inhabitants in 1987 to 1,500,000 inhabitants in 2000 and to about 2,100,000 inhabitants in 2007. However, this population growth has not been monitored by the city planners and decision-makers. Consequently, local authorities have failed to provide neighbourhoods with adequate utilities, services, and infrastructure. Therefore, city-dwellers are facing difficulties, such as getting access to water-supply systems.

*Period of study:* This interdisciplinary research programme was initiated in 2002 with sociodemographic and environmental surveys. However, microbiological and medical investigations were conducted in June 2005 and updated in July 2008 during the rainy season in Yaoundé.

### Data-collection methods

*Target population:* To minimize the risk of confusion between infectious diarrhoea and soft stools normally observed in infants, the study only targeted children aged 6-59 months. Households with no children or whose children did not meet the age criterion were not considered for sampling purposes. In households with several children within this age range, a random age table was used for selecting one single infant.

**Fig. 3.**
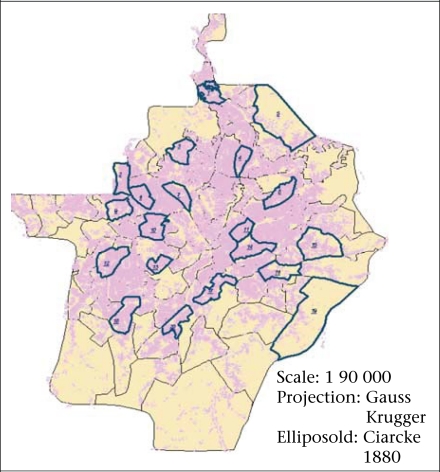
Selected neighbourhoods

**Fig. 4.**
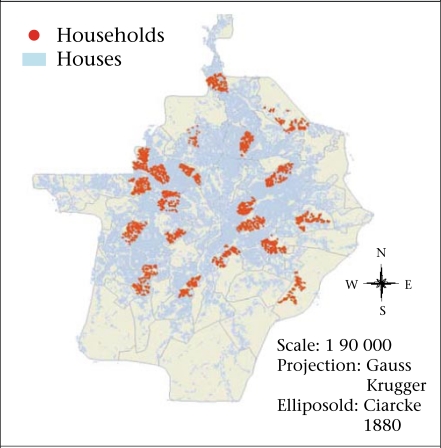
Surveyed households with children aged less than 5 years

*Survey frame and type:* The survey covered neighbourhoods and households in Yaoundé and used a stratified random-sampling procedure based on two stages. First, 20 of the 105 neighbourhoods that make up the city were selected. Not only were these neighbourhoods necessary to derive a sample- size sufficient for the scientific validation of the results but they were representative of the seven types that Yaoundé displays (housing estates, communal plots, wealthy residential neighbourhoods, central spontaneous neighbourhoods, subcentral spontaneous neighbourhoods, urban fringes, and semi-rural neighbourhoods) (Fig. 3). In the second stage of the survey, 3,034 households (Fig. 4) were selected based on having a child aged less than five years as they appear to be more vulnerable to infectious diseases.

Source (of the map): Younede Urban Council Designing: H.B. Nguendo Youngsi, 2009 Software: AreGis 8.2 and Adobe CS 11

Source (of the map): Younede Urban Council Designing: H.B. Nguendo Youngsi, 2009 Software: AreGis 8.2 and Adobe CS 11

A team of the final-year students of the Faculty of Medicine and Biomedical Sciences, and data-collectors from the Cameroonian National Institute of Demography who are specialized in population studies conducted the survey. The team visited selected households to collect data using (a) direct participant-observation technique and (b) structured questionnaire drawn up to respond to the three dimensions of this study, namely:

- The sociodemographic and environmental dimension which aimed at examining sources and methods of collection of drinking-water and storage practices.
- Assessment of the microbiological quality of drinking-water to determine to which extent water used was safe for consumption. In each targeted household, 500 mL of water was sampled in the form usually consumed by inhabitants. Drinking-water stored in bottles or other containers was poured into 500-mL sterile bottles. For pipe-borne water, standpipes were allowed to run for at least one minute and thereafter sanitized before water was aseptically collected in sterile wide-mouth glass bottles. Water from wells and springs was collected in pre-sterilized devices and was then poured in sterile polypropylene bottles. All the samples were labelled with different codes for analysis. The samples were sealed and transported (in an ice-cooled box at about 4°C) to the laboratory and were processed as soon as practicable on the day of collection. Microbiological analyses performed on the samples were total viable count. The total coliforms were enumerated by the membrane filtration (MF) technique described by the American Public Health Association (15). *Salmonella* and *Shigella* species were detected by inoculating water samples into selenite F broth, followed by isolation of the typical organism on selective medium xylose lysine deoxycholate agar (XLD) (16). *Pseudomonas* was detected by placing filtered cellulose nitrate membrane filters onto basic *Pseudomonas* agar. The medium xylose lysine deoxycholate agar was incubated at 42 °C for 40-44 hours. Later, the agar was checked under ultraviolet light to detect pigment thought to be *Pseudomonas aeruginosa* or *Pseudomonas* spp. To detect *Escherichia coli*, all colonies of coliforms, which showed characteristic occurrence on endo agar and mFC agar, were subcultured on eosin methylene blue (EMB) agar, incubated overnight at 37 °C and were subjected to biochemical tests to identify *E. coli* (17). Other enteric bacteria isolated on respective selective or differential media were identified based on their colonial, morphological and biochemical properties, following Bergey's Manual of Determinative Bacteriology (18).
- Approved by the National Ethics Committee of Cameroon, the medical dimension aimed at detecting cases of diarrheoa in children within the selected households. Thus, when a case of diarrheoa was reported, a stool sample was collected and dispatched to the bacteriological, virological and parasitological laboratories of the Cameroon Pasteur Institute within the accepted requirements for the confirmation and identification of the causal agents. Each positive sample was linked with the household's sociodemographic and environmental data.

### Analysis of data

**Fig. 5.**
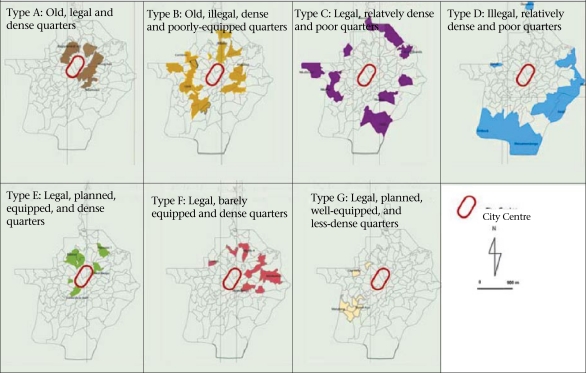
Types of settlements in Yaoundé (for details, see Table 1)

Source: Yoounde Urban Council Production: Nguendo Yongsi, 2006 Software: Adboe Illustrator 11.0 Laboratory: Observatory on Ageing and Society

To have an overview of diarrheoa in the city (spatial analysis), we resorted to modelling. Since the 20 surveyed neighbourhoods were representatives of the 105 neighbourhoods that make up the city, results obtained in the surveyed neighbourhoods were extrapolated to the unsurveyed ones with regard to their respective similarities. This general criterion contained five elements, such as land-occupation modes, geographical situation, quality of housing, level of provision with urban infrastructure, and morphology of the site. The inclusive consideration of those five elements enabled us to elaborate Figure 5, which presents the seven types of settlements. Details on those settlements and examples of both surveyed and unsurveyed neighbourhoods are given in Table 1.

**Table 1.** Types of settlements in Yaoundé according to their spatial organization

Type of settlement	Main characteristics	Neighbourhoods
Housing estates	Legal, recent, planned, less dense, highly provided with water and sanitation infrastructure and with adequate drainage and garbage disposal	Surveyed neighbourhoods: Cité Verte and Biyem Assi
		Unsurveyed ones: Messa, Mballa IV, Mvog Ebanda, Mendong, and Olembé
Communal plots	Legal, dense, barely provided with water and sanitation infrastructure and with adequate drainage and garbage disposa	Surveyed neighbourhood: Elig Edzoa
		Unsurveyed one: Mimboman
Wealthy residential neighbourhoods	Legal, planned, less dense, highly provided with water and sanitation infrastructure and with adequate drainage and garbage disposal	Surveyed neighbourhoods: Nkolndongo 2, Ekoudou Bastos, and Essos
		Unsurveyed ones: Ntem Assi, Nkol Ebogo, Lac, Santa Barbara, and Koweit city
Central spontaneous neighbourhoods	Old, legal, dense, barely provided with water and sanitation infrastructure and with adequate drainage and garbage disposal	Surveyed neighbourhoods: Mvog Ada and Obili
		Unsurveyed ones: Briqueterie, Mvog Mbi, Elig Belibi, and Olézoa
Subcentral spontaneous neighbourhoods	Old, illegal, highly dense, poorly provided with water and sanitation infrastructure and with adequate drainage and garbage disposal	Surveyed neighbourhoods: Mokolo and Elig Effa
		Unsurveyed ones: NgoaEkellé, Oyom Abang, Efoulan, and Ekounou
Urban fringes areas	Illegal, recent, relatively dense, poorly provided with water and sanitation infrastructure and with adequate drainage and garbage disposal	Surveyed neighbourhoods: Emana and Nsam
		Unsurveyed ones: Ekombitié, Nkolmesseng, and Odza
Semi-rural neighbourhoods	Illegal, recent, less dense, poorly provided with water and sanitation infrastructure and with adequate drainage and garbage disposal	Surveyed neighbourhood: Awaé Unsurveyed ones: Simbok, Biteng, and Febe

Based on those observed similarities, the prevalence of diarrhoea recorded in neighbourhoods that were surveyed was then credited to those that were not. The choice of weighted average is justified by the fact that the number of investigated households in surveyed neighbourhoods varied from one neighbourhood to another. This weighted average was obtained by the formula:

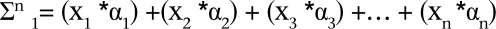

Σ: Standard mean
x_1_: Prevalence of diarrhoea within the first neighbourhood of the considered category
x_2_: Prevalence of diarrhoea within the second neighbourhood of the same category
x_3_: Prevalence of diarrhoea within the third neighbourhood of the considered category, etc.
α: Significance of the surveyed neighbourhood in the category to which it belongs
n : Number of neighbourhoods considered in the n^th^ category neighbourhood.

**Fig. 6.**
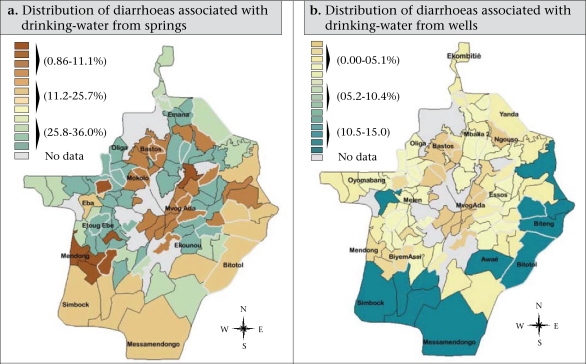
Spatial patterns of diarrhoeas with regard to consumption of groundwater (springs and wells)

**Fig. 7.**
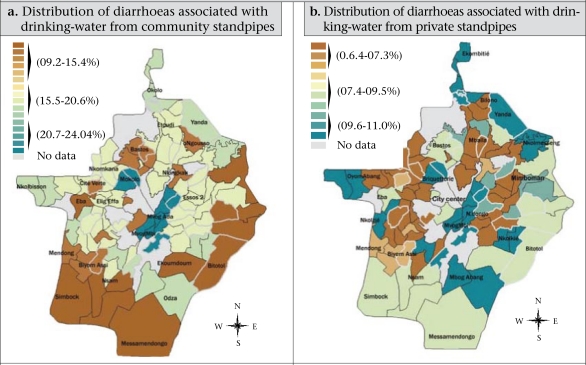
Spatial patterns of diarrhoeas with regard to consumption of groundwater (springs and wells)

This approach permitted to build up Figure 6 and 7. The ArcInfo software (version 8.2) was used for this spatial analysis method whereas the Epi Info software (version 6.04) and the SPSS package (version 11.1) were used for recording and analysis of data. Statistical approaches, such as frequency distribution and chi-square test, were used for analyzing the data, and the p values of <0.05 were considered significant.

Data: PERSAN surveys Classification method: Mean and standard deviation Designng: H.B. Nguendo Youngsi, 2009 Software: Arclnfo 8.2 and ArcView 8.2

Data: PERSAN surveys Classification method: Mean and standard deviation Designing: H.B. Nguendo Youngsi, 2009 Software: Arclnfo 8.2 and ArcView 8.2 Scale: 1 90 000 Projection system: Gauss Krugger Ellipsoid: Clarcke 1880

## RESULTS

### Access to drinking-water in Yaoundé: a differentiated situation

The results of the survey showed that the households in Yaoundé resorted to five different water sources to satisfy their needs (Table 2). In total, 599 (19.7%) households were directly connected to the National Water Company (SNEC), 1,097 (36.1%) shared a common tap located in the courtyard, less than 500 metre away, and 1,042 (35%) fetched water from public taps outside their premises, having to walk a distance of between 500 and 1,000 metre or more.

**Table 2.** Distribution of households in Yaoundé according to drinking-water sources and types of settlements

Type of settlement	Private/individual taps		Semi-private water supply		Community taps		Wells		Springs	
	Frequency	%	Frequency	%	Frequency	%	Frequency	%	Frequency	%
Housing estates	254	8.5	68	2.3	21	0.7	1	0.03	5	0.2
Communal plots	76	2.5	123	4.1	84	2.8	1	0.03	17	0.6
Wealthy residential neighbourhoods	46	1.5	67	2.2	28	0.9	0	0.00	9	0.3
Central spontaneous neighbourhoods	47	1.6	226	7.6	190	6.3	0	0.00	12	0.4
Subcentral spontaneous neighbourhoods	128	4.3	431	14.5	517	17.4	2	0.06	71	2.4
Urban fringes areas	37	1.2	122	4.1	125	4.2	1	0.03	45	1.5
Semi-rural neighbourhoods	11	0.4	60	2.0	77	2.6	4	0.1	66	2.2
Total	599	20.15	1,097	36.9	1,042	35.0	9	0.3	225	7.6

### Microbiological quality of drinking-water in Yaoundé: unsafe water for human consumption

In total, 508 drinking-water samples underwent bacteriological analyses, of which 302 were from households connected to a piped water-supply at home, 154 from wells, 27 from community standpipes, and 25 from springs. From those 508 samples analyzed, 1,242 isolates of enteric bacteria (Enterobactericeae) and 461 isolates of strict aerobic bacteria were obtained and identified. Of the 1,242 isolates of enteric bacteria, 0.2% were *Shigella*, 1.3% *Salmonella*, 5.1% *Escherichia coli*, 12.4% *Enterobacter*, 13.4% *Citrobacter*, 22% *Proteus*, and 37.8% *Klebsiella*. Of the 461 aerobic bacteria, 28.2% were *Acinetobacter,* and 71.8% were *Pseudomonas* (Table 3).

**Table 3.** Distribution of bacterial isolates according to source of drinking-water

Bacteria	Sources of drinking-water							
	Springs		Wells		Community standpipes		Households	
	Frequency	%	Frequency	%	Frequency	%	Frequency	%
Enteric bacteria								
*Citrobacter freundii*	6	24.0	93	60.4	–	–	68	22.52
*Escherichia coli*	7	28.0	29	18.8	–	–	28	9.3
*Klebsiella levinea*	7	28.0	17	11.1	–	–	32	10.6
*Shigella*	1	4.0	1	0.6	–	–	1	0.7
*Enterobacter cloacae*	7	28.0	36	23.4	4	14.8	145	48.0
*Klebsiella pneumoniae*	24	96.0	137	88.9	2	7.4	239	79.1
*Klebsiella oxytoca*	–	–	–	–	1	3.7	–	–
*Serratia*	2	8.0	4	2.6	–	–	8	2.6
*Morganella morgani*	3	12.0	12	7.8	–	–	24	7.9
*Proteus mirabilis*	5	20.0	37	24.0	–	–	73	24.1
*Proteus vulgaris*	3	12.0	53	34.4	–	–	103	34.1
*Providencia*	–	–	4	2.6	–	–	8	2.6
*Salmonella* spp.	1	4.0	5	3.2	–	–	10	3.3
*Enterobacter aerogenes*	–	–	–	–	–	–	1	0.3
*Enterobacter asburiae*	–	–	–	–	–	–	1	0.3
Strict aerobic bacteria								
*Acinetobacter baumannii*	3	12.0	15	9.7	–	–	112	37.1
*Pseudomonas aeroginosa*	12	48.0	57	37.0	2	7.4	147	48.7
*Pseudomonas* spp.	7	28.0	75	48.7	1	3.7	30	9.9

### Health risks associated with consumption of unsafe water

Of the 3,034 children who underwent medical investigation, 437 (14.4%) had diarrhoea). Table 4 shows that the source of water used, which is of poor quality as mentioned above, is critical in controlling diarrhoea.

**Table 4.** Prevalence of diarrhoeal diseases according to drinking-water source

Water source	Frequency	%
Wells	120	27.46
Springs	178	40.73
Households	46	10.52
Community standpipes	93	21.29

Reading key example: Of the 437 diarrhoeal cases, 27.46% were recorded in households using water from wells;

p<0.005, χ^2^ test

#### Spatial disparities associated with consumption of water

With regard to the supply sources, we have noticed an uneven distribution of diarrheal diseases throughout the city (Fig. 6 and 7). Whereas some neighbourhoods were less exposed to diarrhoeas according the supply source used, those were more vulnerable to the disease.

## DISCUSSION

Access to drinking-water in Yaoundé is a worrying situation since 80.2% of the city dwellers did not have access to drinking-water supplied by the national company SNEC. This category of people are those living in the outskirts, such as subcentral spontaneous neighbourhoods and the urban fringes. This is so because, with the galloping population growth, it has been difficult for the SNEC to offer reliable services to everyone. Consequently, many households, not just the poorest, resorted exclusively to groundwaters, such as springs (7.4%) and wells (3%). However, when households that used groundwater as an alternative to avoid high bills or in the case of prolonged shortages/cuts by the SNEC were added, the figure increased to 37.35% for households using water from wells and 63.1% for households using water from springs. Since wells were usually close to houses, households that used water from wells did not suffer much in terms of walking distance (generally less than 50 metre). However, it was quite challenging for those resorting to springs because walking distance varied from 1,000 metre in central spontaneous neighbourhoods to 1,500 metre and even 2,000 metre in the urban fringes and semi-rural neighbourhoods. More intriguing than these figures were water-handling methods, such as collection, transportation, and storage. In fact, many (53.1%) households used uncovered containers, such as buckets, barrels, and PVC basins when collecting drinking-water. The collection procedure consisted of placing containers on the ground and filling them using a plastic pipe held on a leash or in the hands of the user. The plastic pipe which is seldom cleaned is used by everyone and is replaced only in the case of loss or damage. These conditions favour contamination of water with germs more so that collection is done by children (64.9%) who are not very conscious of health risks. Water is then transported in containers laid on the head through a weight-lifting movement during which unclean fingers may be soaked in the water. On the head, in an open container, water is directly in contact with the air and is likely to be contaminated. As far as storage conditions are concerned, many households usually store their drinking-water in uncovered devices, such as buckets (45.5%), clay-pots (33.2%), and barrels (15.4%). This is a less rigorous practice that is conducive to the growth of pathogenic bacteria, which could cause diarrhoea. In some cases (5.9%), water is transferred into plastic bottles. It was noted that the duration of storage varied from one day (15.7%) to three days (47.2%) and even more (22.1%) depending on the size of the household.

Whatever the origin of the water used for consumption (from private standpipes, public taps, wells, or springs), this water is in most of the case unsafe for human consumption. Total bacterial count determined for all the water samples showed that only 25 (4.9%) samples were within the WHO guideline value (<10 cfu/mL) (19). By source, 44.5% of the community standpipes and 100% of the wells and springs samples exceeded the guideline value. Distribution of coliforms across the sources showed that 11.1% of the community standpipes and 100% of the wells, springs, and households exceeded the WHO guideline value of <10 per 100 mL (Table 5).

**Table 5.** Source-wise quality of total bacterial count of water samples

Sample source	% of samples compared with the WHO guideline value		Total number of samples
	Guideline value (<10 cfu/mL)	Excess to guideline value (>10 cfu/mL)	
Households	3.3	96.7	302
Community standpipes	55.5	44.5	27
Wells	0	100	154
Springs	0	100	25

All the samples showed positive results for *Streptococcus* and *P. aeruginosa*. This indicates that the water was not free from faecal contamination as *Streptococcus* is one of the indicators for faecal contamination in drinking-water (20). Although *Pseudomonas* does not harm a healthy individual, it can cause a problem in individual with a weak immune system (21), and it is more reliable and safe if the drinking-water does not show their presence. According to the WHO guideline, total and faecal coliform bacteria should not exceed 10/100 mL in water intended for drinking. The results of the present study clearly indicate that most natural water sources were highly contaminated. It might be due either to poor handling-methods mentioned above, or to the failure of disinfections of raw water at the treatment plant, or to infiltration of contaminated water (sewage) through cross-connection, leakage points, and back-siphonage. However, some studies have associated the occurrence of coliform bacteria in drinking-water system with rainfall events (22). According to these authors, rainfall is a complex variable and may have different impacts on the quality of drinking-water, as rainfall can be a mechanism that introduces coliform bacteria into the system through leaks and cross-connections. Based on both bacterial isolates and total bacterial count performed, the distribution of drinking-water sources according to the microbiological quality is presented in Table 6.

**Table 6.** Distribution of drinking-water sources in Yaoundé according to microbiological assessment

Quality	Sources of drinking-water										Total
	Wells		Springs		Households (sored waters)		Community standpipes				
	Frequency	%	Frequency	%	Frequency	%	Frequency	%	Frequency	%
Safe	0	0.0	0.0	0	10	3.3	15	55.6	25	4.9
Contaminated	154	100	25	100	292	96.7	12	44.4	483	95.1
Total	154	100	25	100	302	100	27	100	508	100

This result is not surprising because the microorganisms found in drinking-water are known to be diarrhoea-causing-pathogens (23,24). Thus, they can be alleged to be the source of diarrhoea diagnosed among children since water used for household consumption is normally collected from wells and springs. This study has also shown a significant neighbourhood-specific geographical variation in childhood diarrhoea (Table 7). In fact, it seems that source of drinking-water supply is also closely related to the type of settlement: neighbourhoods with high exposure to diarrheoa are informal settlements, particularly the spontaneous peri-urban areas. In the category of formal and planned settlements, neighbourhoods of housing estates have been found to be the most vulnerable. Additional insight into these disparities is provided when addressing the phenomenon with regard to the supply sources.

**Table 7.** Prevalence of diarrhoeal diseases according to type of settlements in Yaoundé

Type of settlement	Households surveyed		Diarrhoea cases recorded	
	Frequency	%	Frequency	%
Planned settlement				
Wealthy residential neighbourhoods	158	5.2	12	7.6
Housing estates	352	11.6	31	8.8
Communal plots	304	10.2	41	13.5
Spontaneous settlement				
Semi-rural neighbourhoods	235	7.7	26	13.7
Peri-urban fringes neighbourhoods	342	11.3	57	16.3
Subcentral spontaneous neighbourhoods	1,164	38.3	176	18.4
Central spontaneous neighbourhoods	479	15.7	94	21.6
Aggregate	3,034	100	437	100

p<0.005

*Spatial disparities associated with consumption of water from springs:* A real disparity was observed in the distribution of diarrhoea according to the consumption of water collected from springs (Fig. 6a). The most vulnerable individuals were those who were living in the subcentral spontaneous neighbourhoods, such as Ekounou, Etoug Ebe, and Emana, with a prevalence rate of 25.1-36%. The semi-rural neighbourhoods, such as Eba, Ekombitié, Ahala, and Simbok, and the urban fringes, such as Bilono, Nkolzié, and Oliga, displayed a prevalence rate of 11.2-25.1%.

*Spatial disparities associated with consumption of water from wells:* Figure 6b shows that the neighbourhoods with the high prevalence of diarrhoea were the semi-rural ones, such as Simbock, Biteng, Awaé, and Bitotol, with a prevalence rate of 10.4-15%. These are all semi-rural settings not yet connected to the National Water Company, and accordingly, individuals rely on groundwater for their needs. The less-exposed neighbourhoods were the subcentral spontaneous and fringes neighbourhoods, such as Oliga, Melen, Ekombitié, and Mballa 4 (5.1-10.3%). However, two types of settings were not exposed to diarrhoea. That was the case of the wealthy residential and the central spontaneous neighbourhoods. The situation in the wealthy residential districts was better because those neighbourhoods are inhabited by rich individuals (ministers, members of parliament, and ambassadors) whose houses are all connected to the water company. During the period of water cuts or shortage, they resort to bottled mineral water. Yet in the central spontaneous neighbourhoods, the existing wells were so polluted that individuals themselves found water to be undrinkable.

*Spatial disparities associated with consumption of water from community standpipes (taps):* Several neighbourhoods displayed a high exposure to diarrhoea as the inhabitants resorted principally to the community standpipes for their drinking-water (Fig. 7a). The most exposed were the central spontaneous neighbourhoods, such as Mvog Mbi, Mvog Ada, and Ndamvout, with a prevalence rate of 20.7-24%, and slightly the subcentral spontaneous districts, such as Nsam, Nkomkana, Mimboman, and Elig-Effa, where the prevalence rate ranged from 15.5% to 20.6%. This situation in both the settings might be due to material and financial difficulties which prevent the inhabitants to subscribe private connections from the National Water Company. Since those inhabitants constitute the labour and voting class, the Government has granted them some standpipes.

*Spatial disparities associated with consumption of water from individual/private standpipes (taps):* Throughout the city, only 599 (20.1%) households had access to this water-supply structure. Although these were distributed within the six different urban settings, they were unequally exposed to diarrhoea. Figure 7b shows that the most exposed settings are made up of central spontaneous and of semi-rural neighbourhoods where a prevalence rate of 9.6-1% was recorded. These neighbourhoods are mostly inhabited by low-educated individuals. Being conscious of the frequent and prolonged water rationing or shortages, they regularly stored water in unsafe open containers which permit contamination and from which they drink without any treatment. The less vulnerable were the urban fringes, such as Oliga, Bilono, and Messa-Carrières where a prevalence rate of 6.4-7.4% was recorded.

### Conclusions

The findings of the study point to some important policy implications. For instance, access to safe drinking-water for the majority of urban dwellers requires enabling water regulations that will increase access to drinking-water supplied by the National Water Company. It also suggests paying great attention to water-handling methods by sensitizing households to healthy behaviours in terms of collection and storage conditions. Second, as most individuals use water directly from available sources without any form of treatment, and may, therefore, be exposed to various water-related diseases, it seems logical to suggest that current regulations be revised to include water-quality testing. The control of drinking-water quality in the distribution networks remains a major challenge in sub-Saharan urban areas. However, comprehensive planning should be made for continuous monitoring of water sources, especially the contaminated ones. A further study is needed to determine the factors responsible for the presence of coliforms in drinking-water so that effective intervention can be initiated. As far as possible, water sources must be protected from contamination by human and animal wastes. Third, the geographic information system techniques are useful in assessing health risks concerning population-based studies on drinking-water epidemiology. Data on the uneven distribution of diarrhoea within the city indicate that promotional messages in health education should target the vulnerable neighbourhoods. Of high significance are the most exposed neighbourhoods where efficient and coherent policies should be carried out. It would also be of value to investigate other neighbourhood-level determinants, such as socioeconomic, cultural, environmental and human behavioural factors, involved in the aetiology of diarrheoal diseases.

## ACKNOWLEDGEMENTS

This study was carried out within the PERSAN research programme funded by the Institute of Research for Development. The author is grateful to the staff of the Laboratory of Hygiene and Environment of the Pasteur Institute for their technical assistance. He also thanks the reviewers for their valuable suggestions.
